# A previously low prevalence *Plasmodium falciparum* clone expands in an outbreak in the Pacific Coast of South America

**DOI:** 10.1186/s12879-026-12516-2

**Published:** 2026-01-31

**Authors:** Andrés Cartagenova, Karina Zapata-Berrones, Janeth Boboy, Wilson Cuero, Daniel E. Neafsey, Angela M. Early, Fabián E. Sáenz

**Affiliations:** 1 Centro de Investigación para la Salud en América Latina, Facultad de Ciencias Exactas y Naturales, Pontificia Universidad Católica del Ecuador, Quito, Ecuador; 2 Distrito 08D05 Ministerio de Salud Pública, San Lorenzo, Esmeraldas, Ecuador; 3 Distrito 08D01 Ministerio de Salud Pública, Esmeraldas, Ecuador; 4 Infectious Disease and Microbiome Program, Broad Institute of MIT and Harvard, Cambridge, MA USA; 5 Department of Immunology and Infectious Diseases, Harvard T.H. Chan School of Public Health, Boston, MA USA

**Keywords:** Malaria, Ecuador, Plasmodium falciparum, Genetic diversity, Outbreak

## Abstract

**Background:**

Ecuador is a country working towards elimination of malaria since 2012. The number of malaria cases decreased by more than 99% since 2001. Nevertheless, in recent years the progress has stagnated, and the number of cases has remained constant or increased in some areas. One of the main difficulties for the elimination of malaria in Ecuador is migration from neighboring countries. More than 75% of *Plasmodium falciparum* cases are concentrated in the north coast of the country near the border with Colombia.

**Methods:**

With the goal of contributing to epidemiological surveillance in Ecuador by characterizing the *P. falciparum* parasites circulating in the country, the present study focused on parasites collected in northwest Ecuador to identify the possible origin of *P. falciparum* currently circulating. We collected 72 clinical samples between 2019 and 2021 in three localities of northwest Ecuador as well as samples originating from the bordering Nariño department in Colombia that were diagnosed in Ecuador. We performed an analysis of seven microsatellite markers and whole genome sequencing (WGS) and compared them to previously studied parasites from the region to measure their relatedness as identity by descent (IBD).

**Results:**

Our results show shared, highly clonal parasites between Ecuador and Colombia. All infections were monoclonal. Most importantly, a low frequency clone previously found circulating in the Pacific Coast of Colombia but not in Ecuador caused new outbreaks through clonal expansions. This clone had high genetic differentiation with parasites previously circulating in the Pacific Coast of South America.

**Conclusion:**

This study confirms the persistence of long-term clones in the Pacific Coast of South America and demonstrates the potential importance of low prevalence clones to become epidemic and hamper elimination efforts in low transmission areas.

**Supplementary Information:**

The online version contains supplementary material available at 10.1186/s12879-026-12516-2.

## Background

*Plasmodium falciparum* is the malaria parasite responsible for most deaths. While in South America most cases are caused by *P. vivax*, *P. falciparum* causes considerable burden in the Amazon and along the Pacific Coast [1]. Ecuador has been considered in malaria pre-elimination since 2012 and was part of the malaria E-2020 and E-2025 initiatives (countries that could reach elimination before 2020 and 2025 respectively) [2]. Nevertheless, these goals were not met. In Ecuador more than 75% of *P. falciparum* cases are reported in the northwest part of the country on the border with Colombia. Specifically, the provinces most affected by *P. falciparum* are Esmeraldas and Carchi [3].

**Fig. 1:**
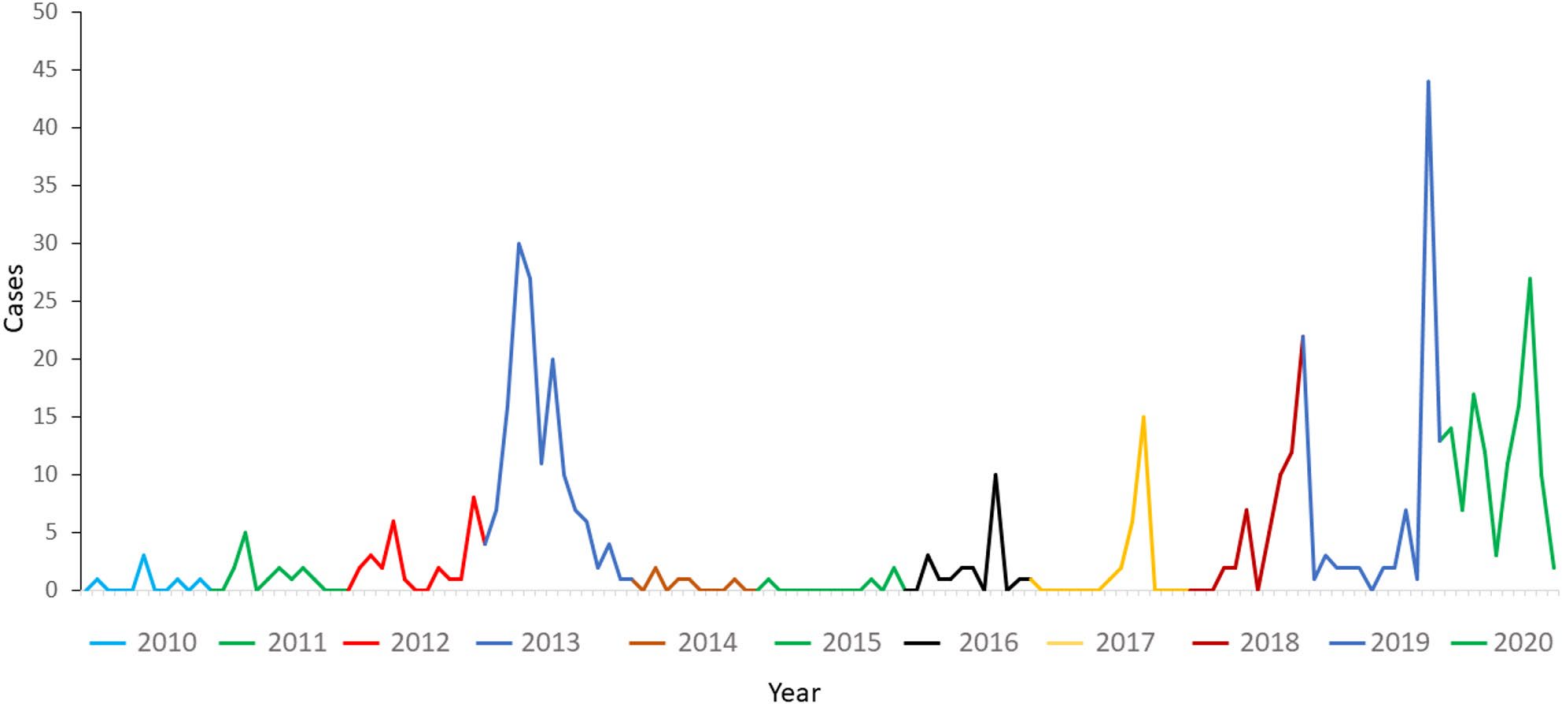
*P. falciparum* cases in Esmeraldas city (2010–2020). Several outbreaks have occurred in Esmeraldas city since 2010. Specifically, the two main outbreaks occurred in 2012–2013 and 2019–2020

The number of *P. falciparum* malaria cases has remained low in the affected areas for the last 15 years (30–200 cases annually from 2012 to 2024). Nevertheless, local outbreaks occur regularly in the north coast of the country and more than 10 malaria outbreaks have occurred in Ecuador in the last 10 years. In 2012–2013, a *P. falciparum* outbreak occurred in Esmeraldas city causing more than 150 cases [4]. Other smaller outbreaks occurred later starting in 2016 with the largest in 2019 (Fig. 1). This outbreak caused 188 reported cases between September 2019 and November 2021 [5].

Population studies of *P. falciparum* have shown that genetic diversity is low in South America when compared to Africa and Asia [6]. On a continental scale, three main genetic populations are observed in South America with little admixture and migration among them (one in the Pacific Coast of Colombia and Ecuador, one in the Guiana Shield and one in the Amazon region of Brazil and Peru) [7]. The Andes represent a natural barrier for the movement of parasites.

The Pacific Coast region of South America is an area of very low transmission [1]. As a result, the *P. falciparum* population in the region is predominated by clonal lineages, some of which have persisted in the region for more than a decade [8]. Relatedness between the clonal lineages is also high [9]. Even though clonal parasites dominate the genetic landscape of malaria parasites, recombination still occurs between different clones and clonal outbreaks represent an important risk factor for the appearance of new clones and are a problem for elimination in the area [10]. Similarly, *P. falciparum* drug resistance mutations are very common in the Pacific coast of South America and drug-resistant genotypes are maintained through time [9, 11].

In Peru, five clones (lineages) were previously defined by microsatellite and SNP typing in 1999–2000 and 2006–2007 [12]. These clones reflect the re-population of Peru by *P. falciparum* after it was almost eliminated. Only one of these clones was found in the Pacific Coast (called clone E) and four (A, B, C and D) were present in the Amazon region of the country [12]. Similarly, in Colombia, distinct clonal lineages have been described using microsatellites [8, 13, 14]. These studies described Colombian parasites from 2008 to 2012 and found they were similar (a match for most markers) to the Peruvian clones B and E and also defined an additional clone F [13]. Several additional clones and recombinants have been described in recent years using both microsatellites and whole-genome sequence data [9]. Particularly, the Amazon of Peru has seen the expansion of the hrp2 negative Bv1 lineage that caused outbreaks in the coast and Amazon [15, 16]. Similarly in a region of the Colombian coast, reduced clonal diversity has been reported and most parasites corresponded to an Ev1 clone originally reported in northwestern Colombia [14, 17].

In Ecuador, a country in elimination, the same pattern of high clonality is present. In 2012–2013 a clonal outbreak occurred in Esmeraldas city. Based on microsatellite markers, the parasites of the outbreak were very similar or identical to the ones circulating in the north coast of Peru in 1999–2000 and the same clone was present in Colombia in 2006 and previously [4, 9]. In addition, these parasites were closely related to the parasite Ecu1110 reported in 1990 [4]. The parasites of northwest Ecuador have been characterized by high clonality and with higher diversity and recombination near the border with Colombia [9, 10, 18].

Recently (2019–2022), a new, larger *P. falciparum* outbreak occurred in Esmeraldas city. A total of 188 cases were reported in the outbreak from 2019 to 2020. With the purpose of contributing to the epidemiological surveillance in Ecuador and characterizing the outbreak that occurred in Ecuador in 2019–2020, the present study characterized parasites collected in three locations: Esmeraldas city, San Lorenzo and Tobar Donoso, which are in Esmeraldas and Carchi provinces respectively, using neutral microsatellite markers and whole genome sequencing.

## Methods

### Ethics statement

Parasite samples used in this study were obtained from the malaria surveillance protocol approved by the Ethical Review Committee of Pontificia Universidad Católica del Ecuador (#CEISH-571-2018) following the declaration of Helsinki. Written informed consent was provided by study participants and/or their legal guardians.

### Sample collection and study sites

**Fig. 2:**
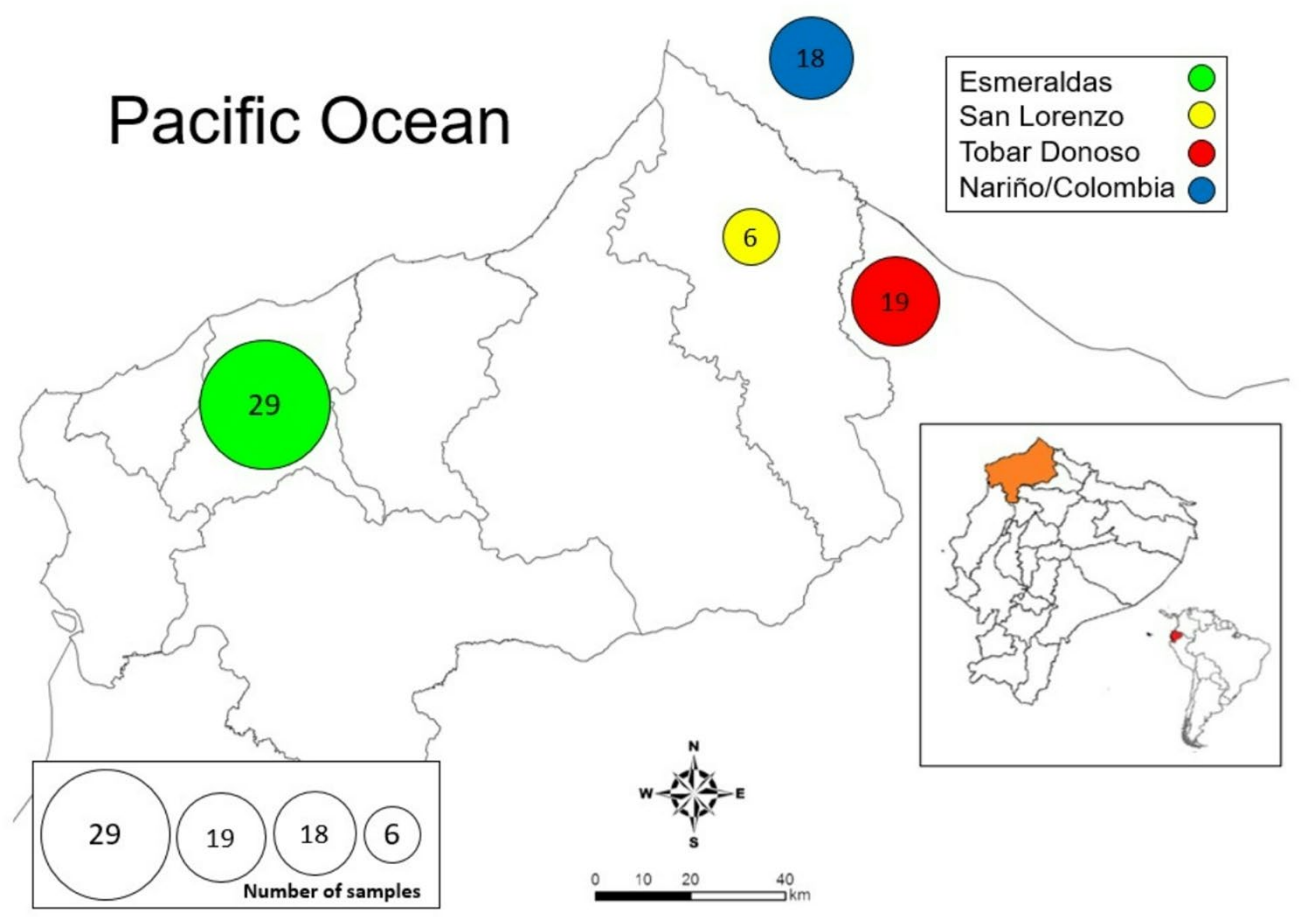
Localities where samples were collected. All samples were collected in four areas shown with a circle containing the number of samples from each locality. Samples from Nariño, Colombia were collected in San Lorenzo but assigned to Nariño according to the travel history of the patient. Each color represents a different locality: Esmeraldas city in green, San Lorenzo in yellow, Tobar Donoso in red and Nariño, Colombia in blue

Samples were collected from patients between March 2019 and September 2021 by the personnel of Esmeraldas and San Lorenzo counties from the Ministry of Health of Ecuador. Seventy-two blood samples (26 samples in 2019, 33 in 2020 and 13 in 2021) were collected from patients who were reported to be microscopically positive for *P. falciparum* infection, and from whom informed consent was obtained. We analyzed samples based on the purported site of infection. For the majority of samples, participants did not report travel history outside the diagnosis area (Esmeraldas (*n* = 29), San Lorenzo (*n* = 6) and Tobar Donoso (*n* = 19)). However, 18 participants who were diagnosed in San Lorenzo reported travel from bordering Nariño, Colombia (Fig. 2). The blood samples were collected by finger prick or by drawing peripheral whole blood and spotted on 3MM Whatman filter paper.

### Reference isolates

In this study, laboratory adapted *P. falciparum* isolates 3D7 and W2 were used as controls for molecular biology studies.

### DNA extraction and confirmation of infection

DNA was isolated from filter paper using the QIAamp DNA mini-spin kit (QIAGEN, Valencia, CA, USA) or Pure Link genomic DNA minikit (Thermo Fisher Scientific, CA, USA). *P. falciparum* infection status was further confirmed by nested PCR using primers for 18 S ribosomal RNA [19], and photo-induced electron transfer (PET) real-time PCR using specific fluorogenic primers for *Plasmodium* (genus) and *falciparum* (species) [20].

### Microsatellite typing

Genomic DNA from all collected samples was genotyped for seven neutral microsatellite loci located in six chromosomes (*TA1*, chromosome 6; *Polyα*, ch. 4; *PfPK2*, ch. 12; *TA109*, ch. 6; and *2490*, ch. 10; *C2M34*, ch. 2; *C3M69*, ch. 3) [6, 21, 22] with fluorescently labelled primers (HEX and FAM) as previously described [6, 12, 21]. PCR products were separated by capillary electrophoresis on an Applied Biosystems 3130xl genetic analyzer (Applied Biosystems Foster City, CA, USA) in Macrogen, South Korea. Alleles were then sized and scored using GeneMapper v3.7 (Applied Biosystems Foster City, CA, USA) and binned to the nearest two or three base pairs. All samples showing results for at least five of the seven microsatellite markers were used for analysis.

### Network analysis

A median-joining network diagram was generated in Network 4.6.1.3 [23] using the seven neutral microsatellites previously described to examine the genetic relationships among the 29 samples collected from the Esmeraldas city outbreak, the samples from San Lorenzo, Tobar Donoso and Nariño and to compare their haplotypes.

### Statistical analysis for microsatellites

Heterozygosity (He) was calculated for four locations (Esmeraldas, San Lorenzo, Tobar Donoso and Nariño) to measure diversity, and pair-wise fixation indices (*F*_*ST*_) were determined for location pairs between Ecuadorian locations and previously characterized *P. falciparum* in the north coast of Ecuador. *F*_*ST*_ values were used to identify similarity between locations and haplotypes. Arlequin 3.5.1.2 software (CMPG, Swiss Institute of Bioinformatics, Lausanne, Switzerland) was used for these analyses [24]. Structure v2.3.4 software [25] was used to assign outbreak samples to previously known groups and Structure Harvester [26] was used to define the number of expected groups. Samples were considered polyclonal by microsatellite analysis if more than one fragment was amplified for at least one marker [12].

### Whole genome sequencing of a portion of the samples

Fifty-four samples underwent selective whole genome amplification at the Harvard School of Public Health [27] before library construction with NEBNext library kits and sequencing at the Broad Institute on an Illumina HiSeqX to generate 150-bp paired-end reads. We combined these newly sequenced samples with 166 monogenomic samples from the South American Pacific Coast originally analyzed in Carrasquilla et al. (2022) [9]. Samples from Carrasquilla et al. (2022) [9] entered our pipeline as unaligned fastq files.

We called variants following the practices established by the Pf3K consortium (https://www.malariagen.net/projects/pf3k). First, we aligned raw reads with BWA-MEM [28] and removed duplicate reads with Picard tools. We jointly called SNPs and indels on the complete set of samples with GATK v3.5 HaplotypeCaller [29]. We performed base and variant recalibration (BQSR and VQSR) steps using a set of Mendellian-validated SNPs. We limited the downstream analysis to variants within the core region of the genome, as defined by Miles et al. [30]. We additionally masked any site that was called as heterozygous in > 10% of samples and masked any individual call supported by fewer than five reads. All samples were estimated to be monogenomic using TheRealMcCoil method [31].

### Identity-by-descent analysis

We analyzed the fractional relatedness between samples as identity by descent (IBD) with the program hmmIBD [32]. For the analysis, we used a set of 20,158 SNPs that excluded all variants within 5 nucleotides of a GATK-identified indel. As described above, all included samples were monogenomic. Our previous work with *P. falciparum* showed that hmmIBD provides reliable fractional IBD estimates for samples with > = 30% of the genome at 5x coverage [33], and so we applied that same threshold to this data set. After IBD calling, we clustered samples using iGraph at a fractional IBD threshold of > 0.99. We visually inspected the assigned clusters for evidence of artifactual cliques, which can result from low quality or contaminated samples, and did not find any, so no further samples were removed from the analysis.

### Datasets used for comparison

Several published datasets have been used to compare the data obtained in this study. In Table 1, we detail the datasets used, their sample years, markers used and citations.

**Table 1:** *Plasmodium falciparum* datasets from Ecuador and Colombia used for comparison with this data obtained in the present study (2019–2021)

Dataset	Sample years	Type (marker used)	Citation
Ecuador	2013–2016	Microsatellites	Vera-Arias et al., 2019
Colombia – CNV	2008–2009		Murillo Solano et al., 2015
Colombia – AN	2010–2012		Dorado et al., 2016
Colombia – Q	2018		Guerra et al., 2022
Ecuador and Colombia	2013–2016	Whole genome sequencing	Carrasquilla et al., 2022

## Results

### Microsatellite profiles

Among the 72 samples collected between March 2019 and November 2021, we obtained results for at least five of the seven markers for 64 samples.

For two of the markers, we found no variation in fragment length across samples. Specifically, TA1 had a size of 171 base pairs and TA109 had a size of 160 bp. These two markers have little variation among the Pacific coast parasites [12].

Heterozygosity (He) was higher in San Lorenzo than in the other two Ecuadorian localities. Esmeraldas had lower He (Table 2). Linkage disequilibrium (IAs) was medium/high for Esmeraldas and Tobar Donoso but low for San Lorenzo and Nariño (Colombia). All samples were monoclonal.

**Table 2:** Heterozygosity (He) and linkage disequilibrium (IAs) of samples in the studied localities

	He	IAs
San Lorenzo	0.314	0.074
Tobar Donoso	0.098	0.241
Esmeraldas	0.0797	0.210
Nariño	0.335	0.0425

When considering results for all seven markers, one complete microsatellite haplotype was found in Esmeraldas city, one in Tobar Donoso, while nine haplotypes were identified for Nariño, Colombia and two haplotypes were found for San Lorenzo (supplementary Fig. 1).

Samples from Esmeraldas city corresponded to a haplotype that was also found in San Lorenzo and in Nariño, Colombia. Samples from Tobar Donoso belonged to a haplotype that was also found in San Lorenzo (supplementary Fig. 1).

When comparing the genetic composition of the different sample sites across time periods, we found that recent samples from Esmeraldas city (2019–2021) appeared differentiated from Esmeraldas city in 2013–2016 (*F*_*ST*_=0.79) and from all other Ecuadorian locations (*F*_*ST*_=0.44–0.82) but less differentiated from Colombian samples collected in Ecuador (*F*_*ST*_=0.26). On the other hand, samples from Tobar Donoso appear highly differentiated from previous and current samples from Esmeraldas (*F*_*ST*_=0.78 and 0.82) but less differentiated from previous samples from the same location (*F*_*ST*_ = 0.27). Samples from San Lorenzo show little differentiation with samples from Nariño, Colombia (*F*_*ST*_=0.030; Table 3). The median-joining network shows that Esmeraldas and Tobar Donoso present a main differentiated haplotype each, while San Lorenzo and Nariño, Colombia have several haplotypes (supplementary Fig. 2).

**Table 3:**
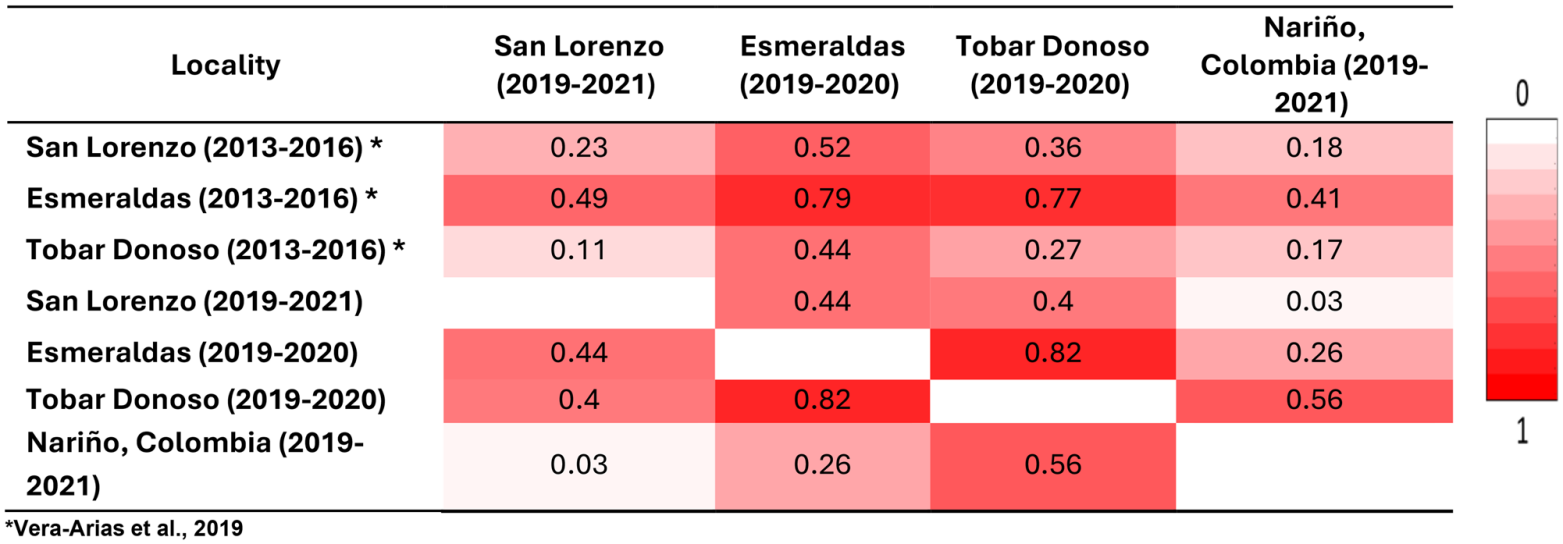
*F*_*ST*_ comparisons between sites from this study (2019–2020) and previously reported samples from the same locations (2013–2016) [18]

### Genetic groups identified by microsatellite profiles

**Fig. 3:**
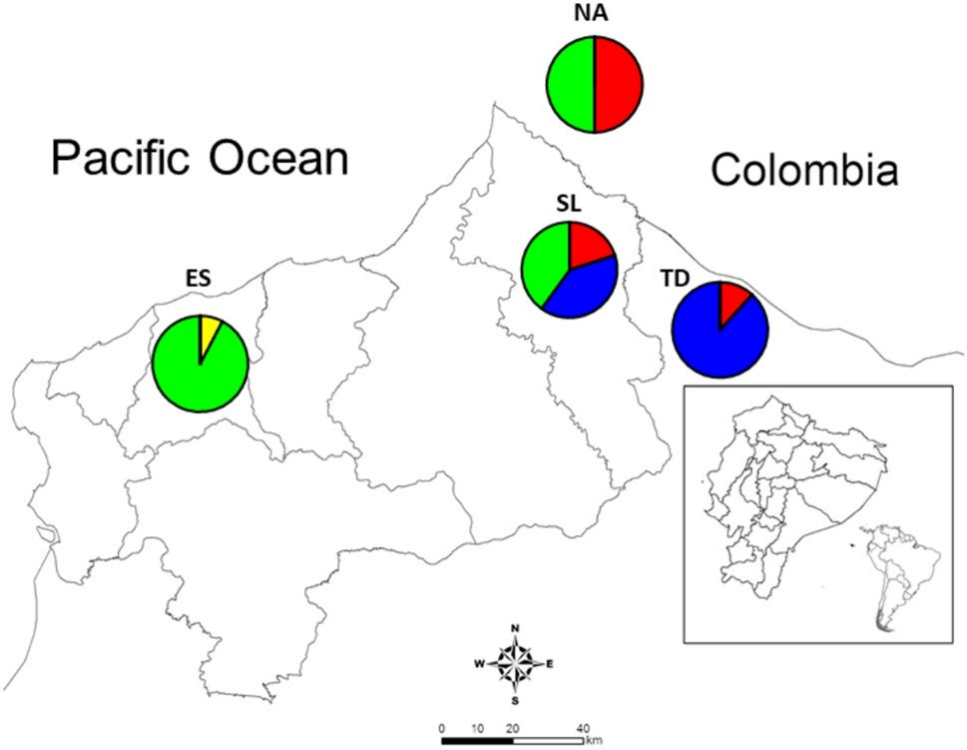
Geographic distribution of *Plasmodium falciparum* genetic groups identified using seven microsatellite markers. Four genetic groups are defined: Two in Esmeraldas (ES), three in San Lorenzo (SL), two in Nariño (NA), Colombia and two in Tobar Donoso (TD)

Using Structure software on microsatellite data, we identified four different genetic groups present in 2019–2020. Most of the isolates from Esmeraldas city corresponded to a unique group not previously reported in Ecuador. The group is also present in San Lorenzo and Nariño, Colombia. On the other hand, most isolates from Tobar Donoso appeared in the same group as previously reported isolates from Esmeraldas city (2013–2016) even though there was a large genetic distance (*F*_*ST*_=0.77). This group is also present in San Lorenzo (Fig. 3). Structure lineages assigned for each of the samples are shown in supplementary Fig. 3.

### Origin of outbreak: comparison between outbreak of 2013 and outbreak of 2019

Twenty-nine samples were analyzed from an outbreak that started the second half of 2019. Twenty-six of the samples had at least five markers reported. All the samples with a complete haplotype (15/26) had the same microsatellite haplotype profile (Table 4). Within this common 2019 profile, five out of seven markers had the same repeat sizes as the single microsatellite profile of the parasites that drove the 2013 Esmeraldas outbreak. Nevertheless, differentiation between the two outbreaks was high according to the analysis (*F*_*ST*_=0.864) (Table 3).

**Table 4:** Complete haplotype of outbreak in Esmeraldas city (2019–2020) and dominant haplotype from previous outbreak in Esmeraldas city (2012–2013) [4]

	TA1	Poly-alpha	PfPK2	TA109	2490	C2M34	C3M69
Esmeraldas city (2019–2020)	171	147	174	160	72	234	124
Esmeraldas city (2012–2013)*	171	147	174	160	72	226	140

The numbers represent the size of the microsatellite amplified fragments. *Saenz et al., 2015

Structure analysis placed the two main haplotypes (Esmeraldas 2013 and Esmeraldas 2019) in two different genetic groups (Fig. 3).

**Fig. 4:**
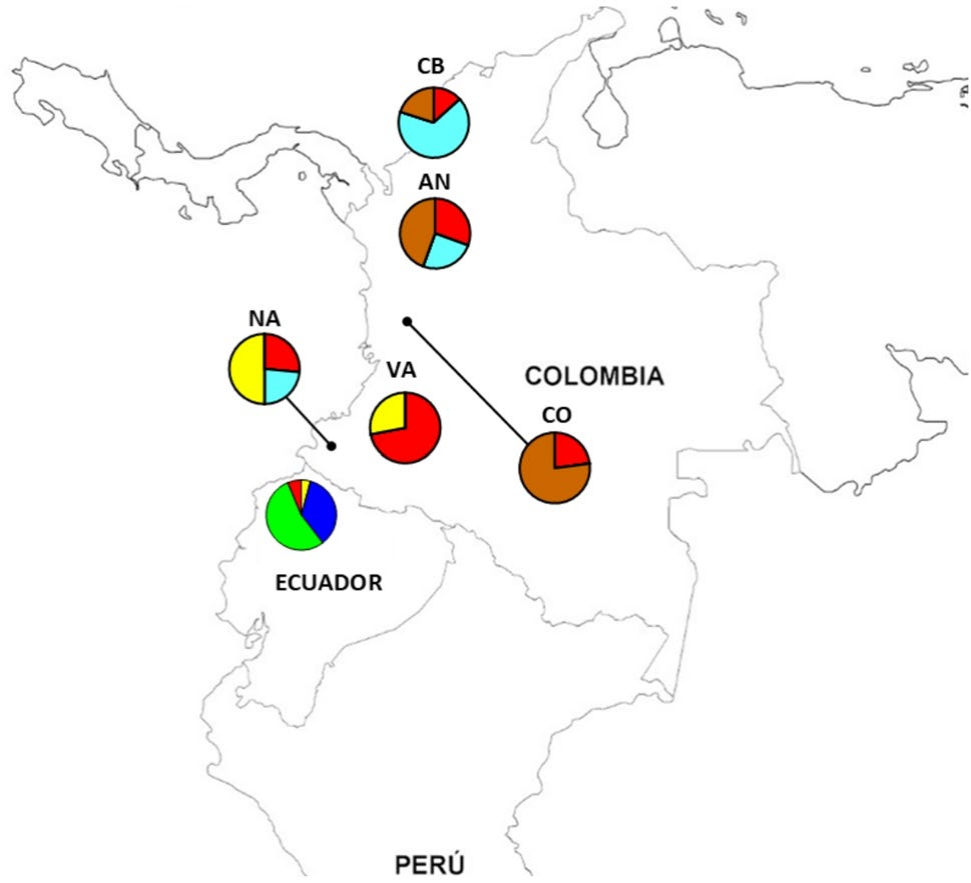
Geographic distribution of *P. falciparum* genetic groups identified using seven microsatellite markers in Ecuador (2019–2020) and Colombia: Cordoba (2008–2009), Nariño (2008–2009), Valle (2008–2009), Antioquia (2008–2012) [13, 14] and Choco (2018) [17]. Six genetic groups were defined (k = 6). The genetic group corresponding to the recent Esmeraldas outbreak is marked in green

When comparing the haplotypes reported for the parasites to previously analyzed isolates from the region, we found that the main haplotype reported in the 2019–2020 Esmeraldas outbreak appears similar in all size fragments to the microsatellite haplotype of isolates from Antioquia, Colombia reported in Dorado et al., 2016 [14] and Guerra et al., 2022 [17] because only one or two base pairs difference occurs in most markers. Nevertheless, Structure analysis placed the two in different genetic groups (Fig. 4). Structure lineages assigned for each of the samples are shown in supplementary Fig. 4.

### Whole-genome analysis of outbreak parasites

In addition to our microsatellite typing, we obtained whole genome sequencing results for 34 isolates: 12 from Esmeraldas city, 14 from Tobar Donoso, 4 from San Lorenzo and 4 from Nariño, Colombia. We analyzed these data alongside 166 whole genome sequence data previously generated for Pacific Coast parasites, including 40 samples from Ecuador [9]. For the full data set, we calculated the relatedness between parasite pairs as identity-by-descent (IBD) and defined “clones” as parasites that were related at an IBD level of 0.99 or above.

**Fig. 5:**
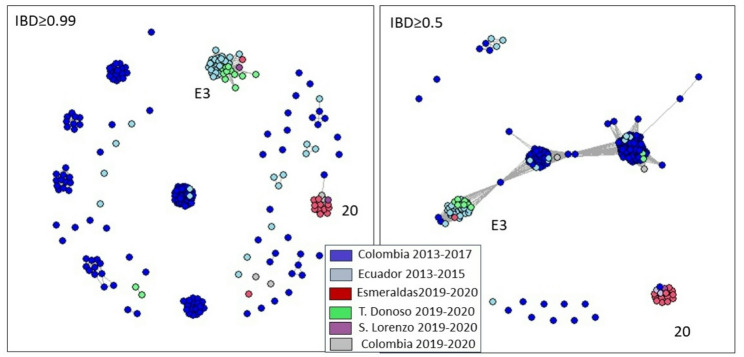
Relatedness networks using pairwise IBD for monoclonal samples from Ecuador and Colombia (2013–2017) combined with samples from northwest Ecuador from this study (2019–2020). The left network shows IBD relatedness higher than 99%. Several clonal groups are identified. The right network shows IBD relatedness higher than 50%. Most samples are connected to the E3 cluster. Most samples (11/12) from the 2019–2020 Esmeraldas outbreak correspond to clonal cluster 20 (as defined in Carrasquilla et al. 2022 [9]) that is unrelated to the rest of the samples. Most samples (12/14) from Tobar Donoso correspond to clonal cluster E3

The whole-genome analysis confirmed the clonal propagation of parasites at all the study sites. Eleven out of twelve sequenced parasites from the 2019–2020 Esmeraldas outbreak correspond to one genetic clone (IBD > 0.99; Fig. 5). We clustered the samples with previously sequenced Pacific Coast parasites and found that the dominant clonal cluster (*N* = 11 samples) was identical to one previously sequenced parasite sampled in Guapi (Cauca), Colombia in 2016 (cluster 20; [9]). The remaining Esmeraldas (2019–2020) sample formed a clonal cluster with all previous samples from the previous Esmeraldas (2012–2013) outbreak (cluster E3). These two clonal clusters are themselves unrelated; they have a very low fractional relatedness (IBD) of 0.032, which is well below the Pacific Coast median of 0.29. These results are concordant with the microsatellite data, which showed that the 2012–2013 and 2019–2020 outbreak clones are distinct. Relatedness between the two outbreak clones and other parasites circulating in the Pacific Coast also varies. The 2012–2013 outbreak clonal cluster (E3) is related at an IBD of 0.5 or greater with multiple other distinct genomes (Fig. 5). However, the 2019–2020 clonal cluster (cluster 20) only shows this level of relatedness with one other genome, which was only sampled once in the population.

Twelve samples out of fourteen from Tobar Donoso (2019–2020) belong to the same clonal cluster that was previously described by Saenz et al. in the 2013 Esmeraldas outbreak [4]. Of the two sequenced samples from San Lorenzo, one belongs to the 2013 Esmeraldas outbreak cluster (cluster E3) and the second belongs to the 2019–2020 Esmeraldas outbreak cluster (cluster 20).

## Discussion

Ecuador has been in a malaria elimination phase since 2012. Nevertheless, initial efforts have stagnated and reaching the final elimination goal has proven more complicated than originally anticipated. This is due in part to the presence of outbreaks that significantly increase the number of cases and may introduce new genomic backgrounds to a population [10, 11].

In this study we confirm that clonal *P. falciparum* lineages constitute a significant portion of the parasites currently circulating in northwest Ecuador. In addition, we confirm that the Pacific Coast of South America represents a single population within which related parasites circulate. The duration of clonal persistence appears to vary between clones. Specifically, the E3 WGS clone (which corresponds to clone E as previously defined by microsatellite profiles [12]) has been present for at least 20 years in Colombia and Ecuador [9] and is still circulating in San Lorenzo, Esmeraldas and Tobar Donoso. On the other hand, more recent clones have expanded in specific areas, as is the case of clone 20 in this work.

**Fig. 6:**
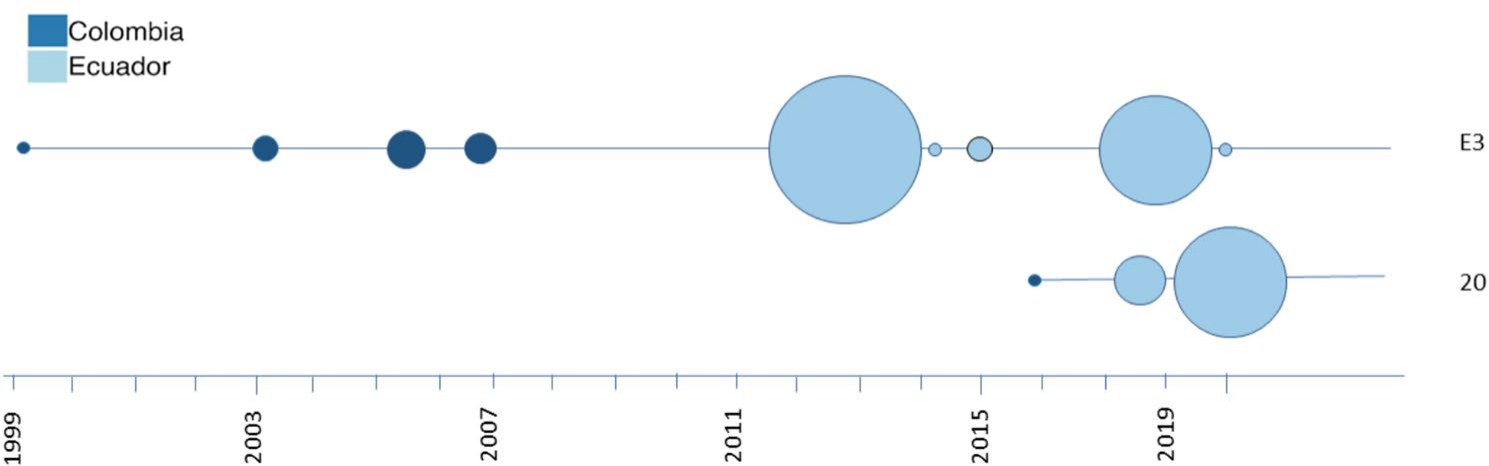
Spatiotemporal distribution of outbreak clones in Ecuador. The clone names were defined in Carrasquilla et al., 2022 [9]. The circle size represents the relative number of samples analyzed in each timepoint

We show that two *P. falciparum* outbreaks in northwest Ecuador have occurred through clonal expansions. A previous outbreak (2012–2013) in Esmeraldas city was the result of a persisting clone in the Pacific Coast of Colombia and Ecuador [4] and possibly in Peru [12]. On the other hand, the more recent 2019–2020 outbreak in the same area comprised a clone detected only once previously in 2016 in Guapi, Colombia (Fig. 6).

It is important to note that by microsatellite markers the clonal cluster 20 samples collected in Esmeraldas city (2019–2020) appear close to the microsatellite-defined Ev1 clone, because they share the fragment size of five of the seven markers and the sizes of the other two are similar in size. Ev1 clone was first described as a related clone to E [14] and found in Quibdo in 2018 [17]. Nevertheless, the use of more advanced analysis techniques argues this possibility. Indeed, our structure analysis on microsatellite results and WGS results suggest that Ev1 reported by Dorado et al., 2016 [14] in Antioquia, Colombia and by Guerra et al., 2022 [17] is not related to the outbreak in Esmeraldas, described in this work.

Nevertheless, we are missing whole genome data for these parasites to determine relatedness between the outbreak parasites described in this study and previously reported Colombian isolates.

While microsatellite data puts the recent Esmeraldas outbreak clone, and clone E [12] close together (only 2 microsatellites differ for most of the samples), WGS data reveals that the parasites causing the recent outbreak in Esmeraldas city are not clonally related to microsatellite-defined clone E [12] and WGS-defined clonal cluster E3 [9]. Rather the 2019–2020 outbreak clone (clonal cluster 20) is highly unrelated to the 2013 outbreak clone (clonal cluster E3; IBD = 0.032). This IBD value is highly atypical among contemporary Pacific Coast parasite genomes, which have a median IBD value of 0.29. This result underlines the importance of the use of powerful techniques such as WGS to better clarify relatedness between isolates.

Because of the presence of an isolate of WGS clonal cluster 20 reported in Guapi, Colombia in 2016 [9], and on both sides of the Ecuador-Colombia border (Nariño, San Lorenzo in April and June 2019) previous to the beginning of the outbreak in Esmeraldas city (in December, 2019), we hypothesize that the clone causing the outbreak originated in Colombia and entered Ecuador through the border through land or maritime migration routes [11]. This finding underlines the importance of cross border malaria research and programs that incorporate endemic regions in both countries, particularly in eliminating areas.

Some of the limitations of this study include the small number of samples reported in this study for some of the sites (in particular San Lorenzo). This is due to the fact that a small number of infections were reported in that location during the study period. In addition, the lack of genomic data for samples reported in previous studies prevents the clarification of relatedness between recent outbreak parasites and previously reported parasites.

## Conclusions

The parasites causing the Esmeraldas outbreak in 2019–2020, described in this study present a genetic lineage not previously reported in Ecuador in addition to resistance gene mutations that have not been previously described in the country [11]. This work therefore indicates that low prevalence genomic backgrounds can cause important outbreaks and that these outbreaks can expand in eliminating areas. Consequently, active diagnosis and surveillance of new infections is necessary in eliminating settings. In addition, more aggressive treatments could be needed to prevent expansion of new clonal infections in eliminating areas.

## Supplementary Information

Below is the link to the electronic supplementary material.

Supplementary Material 1

## Data Availability

Data analyzed in this study is available in CISeAL and can be requested to Dr. Fabián Sáenz. All whole-genome sequence data are available on the NCBI Sequence Read Archive as BioProject PRJNA759192.
