# Supplementary material for: A previously low prevalence Plasmodium falciparum clone expands in an outbreak in the Pacific Coast of South America
Source: BMC Infect Dis. 2026 Jan 31;26:467. doi: 10.1186/s12879-026-12516-2 (PMC12947485; doi:10.1186/s12879-026-12516-2)
Supplement: Supplementary file 1 — Supplementary Material 1 [file 12879_2026_12516_MOESM1_ESM.docx]

**Supplementary material:**


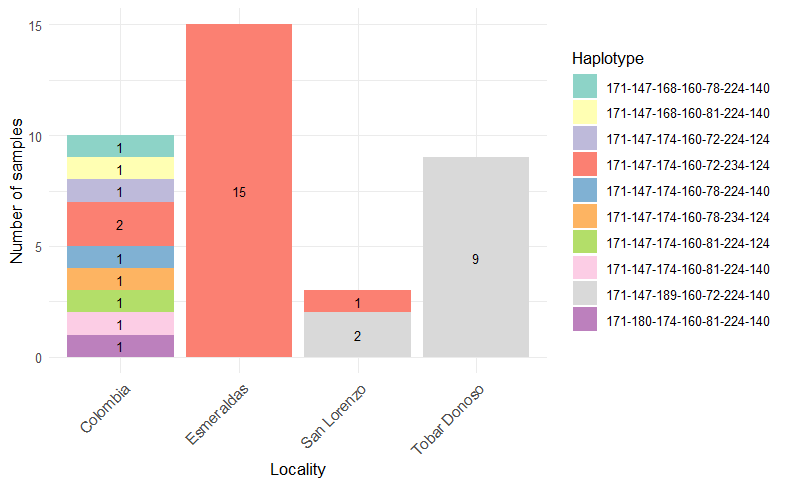


**Supplementary figure 1.** Complete haplotypes identified in this study. Microsatellite markers identified a small number of full haplotypes in Ecuadorian samples.


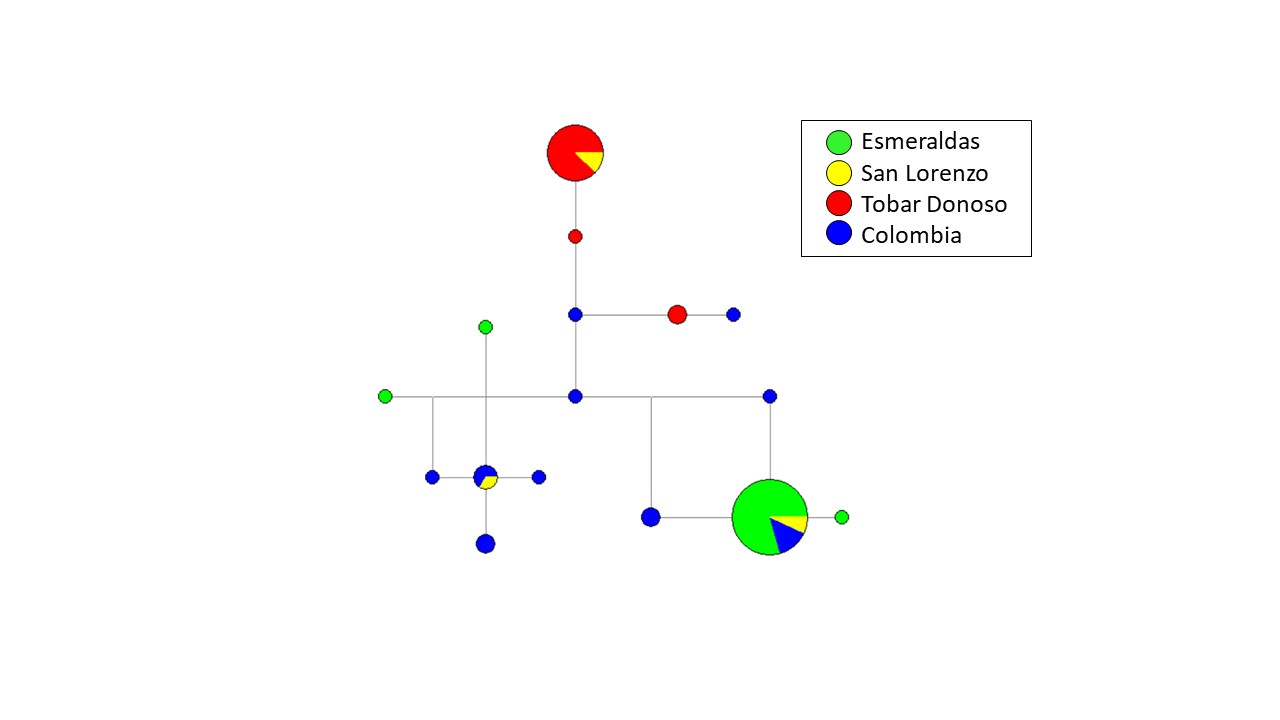


**Supplementary figure 2.** Network analysis of Ecuadorian and Colombian isolates from this study (2019 – 2021). The diagram shows the genotypic relationships among samples from the provinces of Esmeraldas and Carchi (2019 – 2021); Esmeraldas (2019 – 2020), San Lorenzo (2019 – 2021), and Tobar Donoso (2019 – 2020). The dataset also includes Colombian isolates collected in Ecuador between 2019 and 2021. Each color corresponds to a specific locality.

^
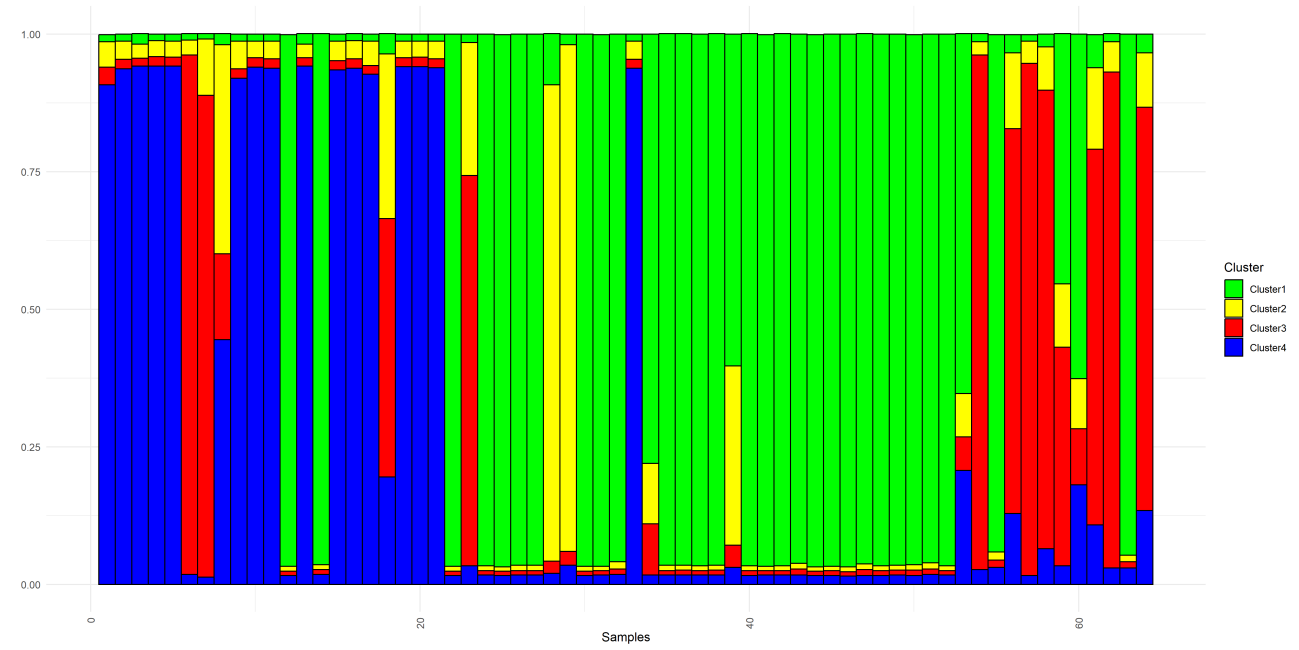
^

**Supplementary figure 3.** Population inference scheme of *Plasmodium falciparum* isolates from Esmeraldas and Carchi provinces, Ecuador (2019 – 2021) using Structure software. The analysis incorporated samples from Tobar Donoso (2019 – 2020; Samples: 0 to 4, 6 to 11, 13, 16, 17, 19 to 21, and 113), Esmeraldas (2019 – 2020; Samples: 25 to 32, and 35 to 52), and San Lorenzo (2019 – 2021; Samples: 5, 12, 15, 22, and 56), as well as Colombian samples collected in the north coast of Ecuador (Samples: 14, 18, 23, 24, 34, 53 to 55, and 57 to 64), between 2019 and 2022. Each color stands for a distinct genetic group.

^
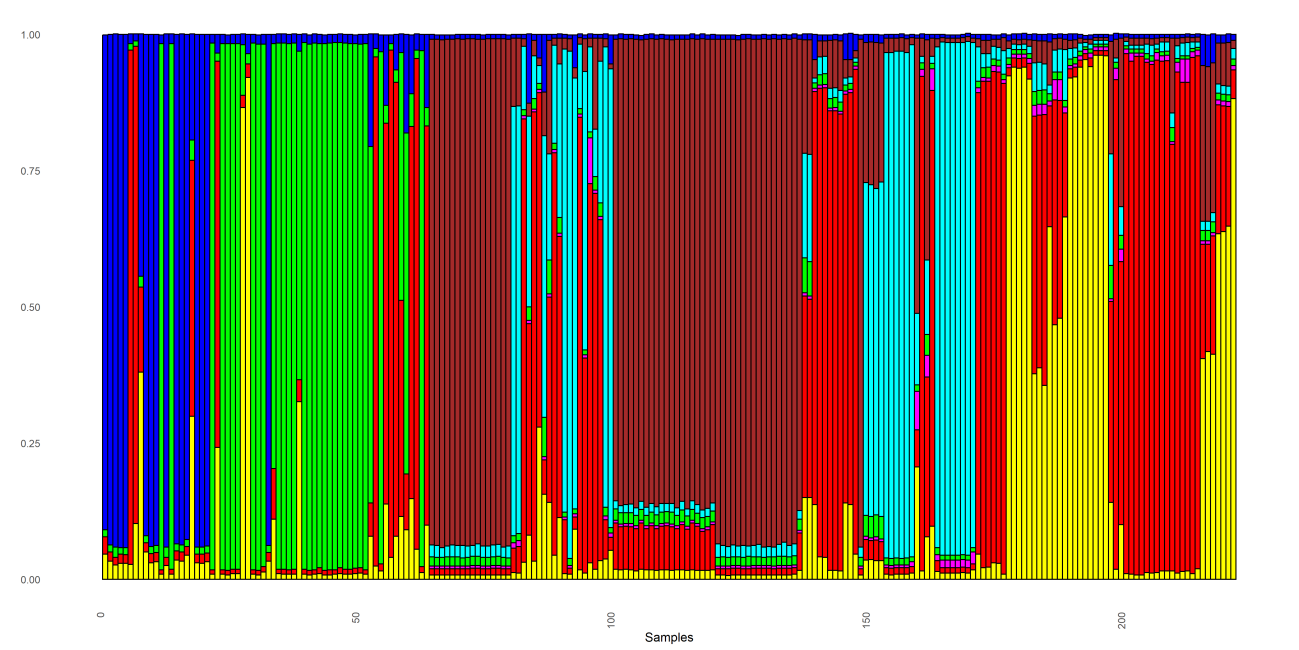
^

**Supplementary figure 4.** Population inference scheme of *Plasmodium falciparum* isolates from Ecuador (2019 – 2021) and Colombia (2008 - 2012 and 2018) using Structure Software. Comparative analyses included Ecuadorian isolates described in Supplementary Figure 2 (Samples 0 to 64), as well as Colombian samples from Antioquia (2012; Samples 65 to 100), Chocó (2018; Samples 101 to 148), Córdoba (2008 – 2009; Samples 149 to 163), Nariño (2008 – 2009; Samples 164 to 197), and Valle (2008 – 2009; Samples 198 to 222). Each color corresponds to a different genetic group.
